# Cognitive and Applied Linguistics Aspects of Using Social Media: The Impact of the Use of Facebook on Developing Writing Skills in Learning English as a Foreign Language

**DOI:** 10.3390/ejihpe10010010

**Published:** 2019-10-03

**Authors:** Blanka Klimova, Marcel Pikhart

**Affiliations:** Department of Applied Linguistics, Faculty of Informatics and Management, University of Hradec Kralove, 530 00 Hradec Kralove, Czech Republic; marcel.pikhart@uhk.cz

**Keywords:** EFL, applied linguistics, learning psychology, educational psychology, foreign languages, cognitive linguistics, psychology and education

## Abstract

Facebook is undoubtedly the most popular social network site nowadays with over two billion users. Therefore, there has been research exploring its potential as a learning environment for various fields of education, including learning English as a foreign language (EFL). As the findings of research studies indicate, Facebook is especially used in developing productive language skills, such as writing, which is considered to be the least popular and the most difficult skill to master. Thus, the purpose of this article is to explore the impact of Facebook on developing writing skills in learning English as a foreign language. The methodology is based on the search for available studies on the research topic, i.e., the impact of Facebook on developing writing skills in EFL, in the world’s databases: Web of Science and Scopus. The search was not limited by any time period. The results of this review article reveal that using Facebook has a positive effect on developing writing skills in EFL classes, especially in shaping and organizing ideas, enhancing motivation, developing and supporting collaboration among peers, improving vocabulary, and reducing students’ shyness. However, more experimental research should be done in this area to reveal other aspects of the writing process, such as communicative language competence, which can be improved by using Facebook, as well as address researchers from continents other than Asia.

## 1. Introduction

Facebook is undoubtedly the most popular social network site nowadays with over two billion users [1]. Therefore, there has been extensive research exploring its potential as a learning environment for various fields of education [2]. Niu’s review study [3] revealed that Facebook appears to be effective as a platform for academic communication, collaboration, information sharing, and enhancing student-centered learning. Furthermore, Lambic [4] found out that students’ performance was positively affected by their use of Facebook. The factor of perceived usefulness also influenced their decision to exploit Facebook as a learning tool. Similarly, Al-Dheleai and Tasir [5] claim that interactions through Facebook can considerably improve students’ academic performance. 

In addition, Facebook enables students to create a supportive learning community among their peers [5,6]. Toker and Baturay [7] show that students with a high grade point average (GPA) and autonomy need Facebook for studying and socialization, and less procrastinating academic works may be highly motivated by the use of Facebook for educational purposes. 

Thus, despite the pitfalls of Facebook, such as the misuse of private data or bullying, it seems to be a promising learning environment for academic purposes, including learning and teaching English as a foreign language (EFL). For instance, in EFL, Facebook can enhance the development of an online community of English language learners where students can practice English language with native speakers [8]. Moreover, experimental studies [9,10] reveal that Facebook may be effective in the enhancement of English language acquisition.

As the findings of Barrot [2] demonstrate, Facebook is especially used in developing productive language skills such as writing, which is considered to be the least popular and the most difficult skill to master [11]. Students are not usually motivated enough to develop the skill of writing. However, Facebook can help with the development of this skills because, as Sakkir et al. [12] and Karimuddin et al. [13] state, students have a positive attitude towards using Facebook in practicing their writing skills. From a cognitive and linguistic point of view, writing is a complex process which comprises other skills, such as lower (remembering and processing) and higher (creating and evaluating) thinking skills [14], as well as communicative language abilities (e.g., linguistic, pragmatic, or discourse competencies) [15].

To conclude, Facebook is a modern tool for communication and can be used as an advanced tool to improve language skills and abilities. When learning a language there are areas which, on one hand, require more attention from the student and, on the other hand, are not considered very interesting, such as writing skills. Therefore, this article attempts to focus on the skill which is usually neglected in language education. Writing skills are somehow considered not very important as they are the least productive in today’s world, which focuses more on the oral aspect of human communication. Paradoxically, it is Facebook which primarily operates as a social media tool, i.e., it is focused on human communication, which can be a useful tool. 

The purpose of this article is to explore the impact of Facebook on developing writing skills in learning English as a foreign language in a view of cognitive and linguistic improvements.

## 2. Materials and Methods 

The authors searched for available studies on this topic in the world’s databases Web of Science and Scopus. The search was not limited by any time period. However, first articles on the use of Facebook and the development of writing skills started to appear in 2011. The authors analyzed and evaluated the findings of the selected studies in order to perform comparison of the findings of the research studies detected on the basis of the following keywords: ‘Facebook AND writing skills in English’ and ‘Facebook AND foreign language learning’.

Altogether, 51 articles were generated from both databases. A total of 27 articles were identified in Scopus and 24 in the Web of Science. After a thorough review of the titles and abstracts, and the duplication of the selected studies, 31 studies were screened and, after that, 23 studies remained for the full-text analysis. Traditional methods of secondary text analysis were used to evaluate the similarities and discrepancies among the texts so that we could conclude what the most important invariable findings of the research were. The secondary text analysis was based on the taxonomical category of contextual research, i.e., lexical relevance and lexical contextual importance. The researched texts were compared and analyzed based on cognitive linguistic approaches, i.e., taxonomized according to their relevance and significance for the outcomes. 

The full-text articles, which provided us with the most important data, were analyzed and evaluated on the basis of the following inclusion and exclusion criteria. 

The inclusion criteria were as follows:

- Only peer-reviewed journal articles written in English were included. This should guarantee a certain quality and relevance of the textual output.
- Only original studies, i.e., empirical studies, were included, so that the data and the results were relevant from an academic point of view.
- The primary outcome concentrated on the impact of Facebook on developing writing skills in learning English. The article wants to highlight the importance and impact of Facebook, mostly because it is the most commonly used app in the world, i.e., it is easily acceptable, and the users will find it easy to use and operate.

The exclusion criteria were as follows:

- The studies which focused on a different research topic, for example, students’ attitude to Facebook in writing classes, were excluded [4,5,6,7,8,9,10,12,13,16,17,18,19,20]. This presented article does not, and cannot, include all the research topics, and it would not be helpful when trying to describe taxonomies which are used when using Facebook in teaching writing skills.
- Descriptive studies and reviews [2,3,21,22] as they are not relevant for the secondary text analysis with the aim of creating learning or linguistic taxonomies.

**Figure 1:**
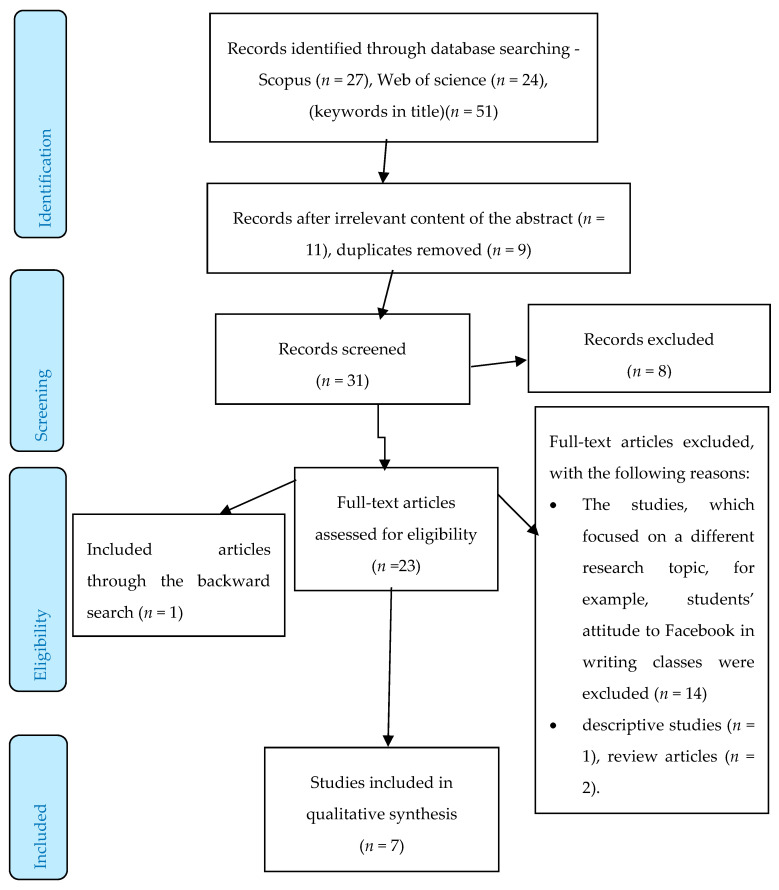
An overview of the selection procedure.

In addition, a backward search was also performed, i.e., references of the detected studies were evaluated for relevant research studies that authors might have missed during their search. This search detected one more article on the research issue. We have, then, covered all relevant sources which should be taken into consideration to analyze the topic thoroughly. Thus, seven studies were eventually analyzed and evaluated. Figure 1 below describes the selection procedure of the detected studies giving the particular details of the subsequent steps in the selection sequence.

## 3. Results

All seven selected studies [23,24,25,26,27,28,29] originated in Asia (i.e., China and Pakistan [26], Indonesia [28,29], Japan [25], Oman [24], Saudi Arabia [23], and Taiwan [27]. The main research topic focused on the effect of using Facebook in developing the skill of writing in EFL classes. Five studies [23,24,25,26,27] were experimental studies with pre-tests and post-tests, one study [28] was an action research study, and one [29] was an observational study. All studies used relevant outcome measures. The research subject samples ranged from 20 to 60 participants, which is a relatively small number, however, still sufficient to come to a relevant valid conclusion. Apart from one study [26], the findings of all other studies indicated positive outcomes of using Facebook in developing writing skills. 

Moreover, linguistic analysis of the given texts proves that the positive results are supported also linguistically. All the researched texts bring important outcomes regarding the use of Facebook in the respondents or researched groups. In addition, all the students who used Facebook when improving their writing skills seemed more activated and motivated to learn this skill, so that the learning process was enhanced by the tool, which is often considered as a mere social media tool. They also enhanced their collaboration by using Facebook dramatically. Table 1 below summarizes the key findings of the selected studies, which are ordered alphabetically according to the surname of their first author.

## 4. Discussion

The findings of this review article reveal that using Facebook has a positive effect on developing writing skills in EFL classes. This is in line with other research studies on this topic (e.g., [2,18,22]). It especially contributes to shaping and organizing ideas when writing [27]. Using Facebook can also improve vocabulary [23], which can be achieved through exposure to fellow group members’ posts [16]. Furthermore, using Facebook can reduce students’ shyness [26] or lower self-esteem [12], particularly when students feel comfortable with each other, and are supported by each other, when developing writing skills [5,6,21]. Improving vocabulary is one of the most important areas in learning a new language, and a supportive environment with improved social skills is crucial from the viewpoint of mental development. From an applied linguistics point of view, using Facebook is a powerful tool to enhance cognitive and productive skills in language educational process, mostly in the EFL where there is a need for simple accessibility of learning materials. 

In addition, Facebook is the most popular social network sites among students, and they use it daily, which makes it an easily accessible tool for everyday contact with the target language. The fact that they are in everyday contact with Facebook makes them quite motivated to use it also in formal settings such as in EFL writing classes [22,26,27,28]. The findings of the described research studies, however, indicate that the most suitable approach to learning and teaching writing skills is a blended learning approach, which combines both traditional, face-to-face teaching with Facebook learning [13,28]. Blended learning has proven to be a very efficient tool and combines both traditional methods of school education with modern means of communication and e-Learning. Facebook then connects traditional approaches with modern ones, enabling the students to use modern technologies and stay in touch with peers. This benefit is very important from a psychological viewpoint, as the influence of peers is a crucial social and psychological phenomenon for the young generation. 

Interestingly, the demographics illustrated that all studies were published in Asia. Barrot [2] came to the same conclusion. However, in comparison with Barrot [2], who in his review detected only one experimental study when exploring all four language skills, the authors of this review article identified five experimental studies focused on only one language skill, namely writing. This signifies that research in this area is on the rise. This paper highlights the importance of the possibilities of Facebook in learning English and, moreover, it attempts to underline the importance of the topic from a taxonomical point of view. Facebook has a chance to improve cognitive skills (mostly those which are generally described by the term ‘writing skills’), and there are not very many easily accessible devices for this particular language competence. Obviously, there are many other social media platforms which could have a similar effect on the improvement of language competence in second language acquisition—however, this paper is limited to the utilization of Facebook mostly due to its ubiquity and familiarity among the younger generation. 

The limitations of this review article include a relatively small number of studies found on the research topic, as well as different methodologies used in the detected non-experimental studies and slightly small subject samples in all selected studies, which might contribute to the overestimation of the described findings. However, the authors claim that the conducted analysis has led to proper generalization and that the results have validity which can be transferred into further linguistic and psychological research in this area. Furthermore, it can be a stimulus for further research into the use of electronic devices in cultivating and improving particular language competencies, such as reading, speaking, writing, and listening. The vast possibilities of social media, mostly for the younger generation, are still to be researched and this paper is an attempt to compare and analyze previous results bringing together core results, and creating a synergy between them. It is also very important to continue the research into the negative impact of social media, not only from a psychological point of view, but also from the perspective of learning psychology and education.

## 5. Conclusions

Overall, Facebook appears to be a promising and flexible tool with the potential to enhance and improve second language writing skills as the results of this review study have shown. However, more experimental research should be performed in this area to reveal other aspects of the writing process, such as linguistic competence, which can be improved by using Facebook, as well as address researchers from continents other than Asia. 

The paper attempts to show, by comparing and analyzing the particular findings of scarce results, the possibilities and opportunities that Facebook brings to the learning process, namely with respect to ESL and writing skills. It has been confirmed that Facebook will bring a new outlook to the learning process by enhancing student participation and will potentially bring further new approaches to the learning process. The use of Facebook has a positive effect on developing writing skills in English as a foreign language, especially in shaping and organizing ideas, enhancing motivation, developing and supporting collaboration among peers, improving vocabulary, and reducing students´ shyness. 

Obviously, there are other social media which are used a lot—however, we claim that, due to the fact that Facebook is the most popular and easily accessible social media platform, it is easy to leverage its popularity and implement it in language education. Further research into this area will naturally bring a lot of important findings so as to optimize language education and particularly second foreign language acquisition.

Learning a foreign language is a process which takes a long time, and it can be demotivating for the participants. Therefore, any methodology which makes the process smoother and easier should be accepted as it improves the outcomes of the learning process and the satisfaction of the participants. 

## Figures and Tables

**Table 1:** An overview of the findings from the selected studies.

Study	Objective	Characteristics of subjects and intervention period	Outcome measures	Findings
Ahmed [23]	To explore the effect of Facebook on grammar discussions and writing competencies in English.	60 undergraduate university female students (30 in the experimental group and 30 in the control group). The intervention lasted from January till May 2016 and students in the experimental group were using Facebook, while the control group was taught in a traditional way.	Pre- and post-tests, statistical analysis.	The experimental group outperformed the control group in grammar and writing.
Alkhoudary [24]	To investigate the effect of using Facebook in EFL writing classrooms.	60 university female students (30 in the experimental group and 30 in the control group) and 30 teachers (15 in the experimental group and 15 in the control group). The intervention period lasted for one semester and included using Facebook in the experimental group, while the control group was taught in a traditional way.	Questionnaires, interviews, pre- and post-tests, statistical analysis.	The experimental group outperformed the control group in grammar and writing. In addition, they improved their vocabulary and collaboration with their peers.
Dizon [25]	To examine the effect of using Facebook in the enhancement of L2 writing.	30 Japanese university English undergraduate students, 16 in the experimental group and 14 in the control group. The intervention, consisting of using Facebook or pen and paper when writing, lasted for 12 weeks.	Three continuous assessments, statistical analysis.	The experimental group made more significant gains in terms of writing fluency, but neither group significantly improved in terms of vocabulary and grammatical accuracy.
Khan et al. [26]	To explore Facebook effect on EFL learners’ writing approach.	20 university students (10 in the experimental group and 10 in the control group). The intervention period was not specified in the article, it included Facebook instruction for the experimental group and traditional teaching for the control group.	Pre- and post-tests, statistical analysis.	No differences between using Facebook and traditional teaching methods. However, using Facebook reduces students’ shyness.
Shih [27]	To investigate the impact of the use of Facebook and peer assessment in blended English writing course.	23 first-year students majoring in English. The intervention period, lasting for 18 months, included one-third of a semester of classroom instruction and two-thirds of a semester combining Facebook, peer assessment, and classroom instruction.	Pre-test and post-test of English writing skills, a self-developed survey questionnaire, in-depth student interviews, statistical analysis.	The results reveal that students improved their English writing competencies. In addition, the use of Facebook can considerably stimulate students to learn.
Sulisworo et al. [28]	To explore the effectiveness of blended mobile learning activity with the help of Facebook to enhance student writing competencies.	61 undergraduate students (13 males and 48 females). The intervention, combining classroom learning and using Facebook, lasted for 12 weeks, each session per week for 100 minutes.	Essay scoring.	By using a blended learning approach, students particularly enhanced the skill of shaping ideas and organizing their ideas into written form, in addition to becoming more active.
Tahir and Suriaman [29]	To examine the impact of exploiting Facebook in the enhancement of English writing narrative text.	47 second-year students. The intervention included eight cycles and each cycle consisted of five 45-minute lessons.	Observation, test, statistical analysis.	Writing a narrative text using Facebook can enhance students’ writing skills and make students motivated in learning.

## References

[1] (2019). Top 15 Most Popular Social Networking Sites and Apps. https://www.dreamgrow.com/top-15-most-popular-social-networking-sites/.

[2] Barrot J.S. (2018). Facebook as a Learning Environment for Language Teaching and Learning: A Critical Analysis of the Literature from 2010 to 2017. J. Compu. Assist. Learn..

[3] Niu L. (2019). Using Facebook for Academic Purposes: Current Literature and Directions for Future Research. J. Educ. Comput. Res..

[4] Lambic D. (2016). Correlation between Facebook Use for Educational Purposes and Academic Performance of Students. Comput. Hum. Behav..

[5] Al-Dheleai Y.M., Tasir Z. (2017). Using Facebook for the Purpose of Students’ Interaction and its Correlation with Students’ Academic Performance. TOJET.

[6] Ali A. (2016). Medical Students’ Use of Facebook for Educational Purposes. Perspect. Med. Educ..

[7] Toker S., Baturay M.H. (2019). What Foresees College Students’ Tendency to Use Facebook for Diverse Educational Purposes?. Int. J. Educ. Technol. High. Educ..

[8] Kabilan M., Ahmad N., Abidin M. (2010). Facebook: An Online Environment for Learning of English in Institutions of Higher Education?. Internet High. Educ..

[9] Sim M.A., Pop A.M. (2014). The Impact of Scial Media on Vocabulary Learning, Case Study—Facebook. Ann. Univ. Oradea Econ. Sci. Ser..

[10] Khoshnoud K., Karbalaei A. (2014). The Effect of Interaction through Social Networks Sites on Learning English in Iranian EFL Context. J. Adv. Engl. Lang. Teach..

[11] Klimova B. (2015). A Summary Approach to the Teaching of Writing. Proceedings of the INTED 2015 Conference.

[12] Sakkir G., Rahman Q., Salija K. (2016). Students’ Perception on Social Media in Writing Class at STKIP Muhammadiyah Rappang, Indonesia. Int. J. Engl. Linguist..

[13] Karimuddin, Nasiruddin, Haryanto, Rahman A. (2015). EFL Student’s Perception towards Using Mobile Technology via Facebook for Practicing English Writing Skill: A Revolution of Traditional to Modern Teaching. J. Lang. Lit..

[14] Bloom B. (1956). Taxonomy of Educational Objectives, Handbook 1: The Cognitive Domain.

[15] Frydrychova Klimova B. (2012). Teaching Formal Written English.

[16] Bani-Hani N., Al-Sobh M., Abu-Melhim A.R. (2014). Utilizing Facebook Groups in Teaching Writing: Jordanian EFL Students’ Perceptions and Attitudes. Int. J. Engl. Linguist..

[17] Dizon G. (2017). Facebook vs. Paper-and-Pencil Writing: Comparing Japanese EFL Students’ Opinions of the Writing Mediums. Lang. Teach..

[18] Saeed M., Abdulrab M., Ghazali K., Sahuri S. (2018). Engaging EFL Learners in Online Peer Feedback on Writing: What Does It Tell Us?. J. Inf. Technol. Educ. Res..

[19] Yu L.T. (2018). Incorporating Facebook into an EFL Writing Course: Student perception and Participation in Online Discussion. CALL-EJ.

[20] Vikneswaran T., Krishnasamy P.K. (2015). Utilising Social Networking Sites to Improve Writing: A Case Study with Chinese Students in Malaysia. Technol. Pedagog. Educ..

[21] Selcuk H. (2017). Peer Affective Factors in Peer Collaboration: Facebook-Based Collaborative Writing Activity among Turkish High School EFL Learners. Tomorrow’s Learning: Involving Everyone, Proceedings of the Learning with and about Technologies and Computing, WCCE 2017, Dublin, Ireland, 3–6 July 2017.

[22] Ramadhani P. (2018). Using Facebook Comments in Teaching Writing Skill. Proc. ICECRS Conf..

[23] Ahmed M.A.E.A.S. (2016). Using Facebook to Develop Grammar Discussion and Writing Skills in English as a Foreign Language for University Students. Sino-US Engl. Teach..

[24] Alkhoudary Y.A.M. (2018). Utilizing Facebook in EFL Writing Classrooms in Oman. IJCDS J..

[25] Dizon G. (2016). A Comparative Study of Facebook vs. Paper-and-Pencil Writing to Improve L2 Writing Skills. Comput. Assist. Lang. Learn..

[26] Khan M.S., Ayaz M., Khan S., Shah S. (2016). Facebook Effect on Enhancement of English Learners’ Writing Approach at University Level in Khyber Pakhtunkhwa. J. Lit. Lang. Linguist..

[27] Shih R.C. (2011). Can Web 2.0 Technology Assist College Students in Learning English Writing? Integrating “Facebook” and Peer Assessment with Blended Learning. Australas. J. Educ. Technol..

[28] Sulisworo D., Rahayu T., Akhsan R.N. (2016). The Students’ Academic Writing Skill after Implementing Blended Learning Using Facebook. Inf. Technol. Learn. Tools.

[29] Tahir S.Z.B., Suriaman A. Improving Students’ Writing Skill through Facebook at University of Iqra Buru. https://www.researchgate.net/publication/324571542_IMPROVING_STUDENTS%27_WRITING_SKILL_THROUGH_FACEBOOK_AT_UNIVERSITY_OF_IQRA_BURU.

